# Supplementary material for: Direct and moderating effects of environmental regulation intensity on enterprise technological innovation: The case of China
Source: PLoS One. 2019 Oct 7;14(10):e0223175. doi: 10.1371/journal.pone.0223175 (PMC6779245; doi:10.1371/journal.pone.0223175)
Supplement: Attachment — Submitted filename: Response to reviewers.docx [file pone.0223175.s001.docx]

Comments:

Reviewer #1:

The paper is interesting and relevant but needs more work.

1. The abstract is too long but not informative.

2. The paper needs language editing.

3. The development of the literature needs more work to link them clearly.

4. The review also needs more recent works.

5. Tables and figures need to be more professionally presented.

6. Table 2 has an R2 of 90+ % is that acceptable or there is some issue of tautology.

7. Discussion needs more work.

8. Implications need to be more specific.

9. There should be some limitations and suggestions for future research.

Reviewer #2:

Direct and Moderating Effects of Environmental Regulation Intensity on Enterprise Technological Innovation, it is an important study theme covering different countries and regions all over the world. In this sense, taking the the industrial enterprises with an annual turnover of over 20 million yuan from 30 Chinese provinces or municipalities between 2008 and 2016 in China as an example to explore the Direct and Moderating Effects of Environmental Regulation Intensity on Enterprise Technological Innovation with a panel data regression model does make sense. However, this study did not gain the expected intriguing results or findings and greatly contribute to the previous literature. Thus, I have the following concrete comments:

The literature review has not clearly presented the gap within the current literature, as a result, it is very hard for me to evaluate this paper’s theoretical contribution to the discipline. I do hope the authors could improve this term greatly in the further version.

Totally, the mainly part of the paper brings “little nutrition” to the extension of the research in this field.

In the conclusion, if the author can make a comparative analysis of the research results with previous studies or related studies, it may be helpful for the deepening of this paper.

Reviewer #3:

I am afraid that this article is not publishable in PLOS One at current form. There are a few significant issues that require fundamental rethinking.

First and the utmost, the claimed contribution of the paper is not convincing; adding or changing variables do not necessarily imply for added-value to the literature of “Porter hypothesis.” In particular, the indicator of “government financial support” – the government-financed fund in R&D expense – does not make sense since the overall funding can not distinguish whether it is for environmental-friendly technologies or not. When you put this variable in a regression model of technological innovation, which is also not referring to green technologies but the overall innovation efforts, the positive coefficient estimates have very little implication. Take the material industries, for instance, Ministry of Science and Technology released the “13th Five-Year-Plan for S&T Innovation of Materials Industries” in 2017, which proposed to invest substantially on new materials. However, there is only a minimal investment in environmental-friendly materials. Obviously, the model does not work for these industries. The fact is that public R&D investments of green technologies for many other sectors in China are also little. To evaluate the synthetical effect of environmental regulation and public R&D supports on green innovation, singling out public funding for green technologies is necessary, although the data might not be available in all the yearbooks.

Secondly, there are a lot of similar researches published in Chinese peer-review journals in the past five years or so. Many of those articles used the same databases and related variables, however, it’s necessary to have some critical thinkings to push forward the discussion of “Porter hypothesis” in China.

At last, this article is a standard and textbook research in innovation study, which can not be accounted for as interdisciplinary research that PLOS One’s readers are expecting to.

Dear Editor and Reviewers:

Thank you for your letter and for the Reviewers’ comments concerning our manuscript ID PONE-D-19-15132 entitled “Direct and Moderating Effects of Environmental Regulation Intensity on Enterprise Technological Innovation: The Case of China”. These comments were all valuable and very helpful for revising and improving our paper. We have studied the comments carefully and made corrections that we hope will be met with approval. The revised portions are shown in red in our manuscript. The main corrections in the manuscript and the response to the reviewers’ comments are shown as follows.

Response to Reviewer 1 Comments:

Thanks a lot for your encouragement. We greatly appreciate your time and very professional and helpful comments. We have carefully read some new literature related to environmental regulation and added Professor T. Ramayah’s research works on green human resource management in recent years. We have also checked our writing. More details can be found in the revised portions, which are shown in red in the manuscript. Our responses are provided as follows.

Answer (1): Thank you for your valuable questions and professional suggestions. Following your valuable suggestions, we have re-edited the abstract and added a graphical abstract to the manuscript. The abstract is shown as follows:

There is few significant attempt to integrate environmental regulation, government financial support, and corporate technological innovation in a methodological framework. Employing the data of the industrial enterprises with an annual turnover of over 20 million yuan from 30 Chinese provinces or municipalities between 2008 and 2016, this paper applies the fixed effect regression model to reveal the relationships between environmental regulation, government financial support, and corporate technological innovation simultaneously. Results show that: (1) there exists a U-shaped relation between environmental regulation intensity and technological innovation of enterprises which declines first and then climbs up, and China is still at the stage of inhibition before the “inflection point”. (2) government financial support does not significantly work on technological innovation directly, but environmental regulation drives this effect to be achieved; when the value of ln*ER* is higher than 3.69, government financial support can significantly facilitate corporate technological innovation. (3) the comparison between regional samples reveals that heterogeneity exists in the influence of environmental regulation intensity and government financial support on corporate technological innovation. The threshold value of enabling effects of environmental regulation in eastern region is higher than that of the central and western region. These results remain consistent after we experiment several robustness checks. Theory and policy implications of our work are discussed.

The graphical abstract is shown as below:

Environmental regulation intensity

Environmental regulation intensity

Direct effects
(U-shaped relation)

The linear moderating effects

(significantly)

Government financial support

Environmental regulation intensity

Enterprise technological innovation

Environmental regulation intensity

Direct effects
(not significantly)

)

Answer (2): Thanks a lot for your careful readings and professional suggestions. Following your valuable suggestions, we have corrected our mistake and tried our best to carefully check the writing of this paper. Moreover, the revised manuscript has been edited by the native speakers.

Answer (3): Thank you for your professional suggestions. Following your valuable suggestions, we have re-edited literature review to link the development skeleton of the literature clearly. Which is benefit for presenting the gap within the current literature. The literature review is shown as follows:

Our article relates to two main strands of the literature. The first category of literature concentrates on environmental regulation’s effects on enterprise technological innovation, and it was first mentioned by Magat (1978) who believes that innovation is the key to dissolve the contradiction between environmental protection and economic development. But opinions on the relationship between these two diverge. On the one hand, traditional neoclassical theory holds that environmental regulation can improve social benefits on the whole but will inevitably increase the costs of enterprises, lowering their capability in technological innovation. This constitutes the “compliance costs” entailed by environmental regulation. Gray (1987) reveals, based on the data from American manufacturing and power industry, the negative effects of environmental regulation on innovation level and economic growth as environmental regulation would increase enterprises’ costs in emission reduction and lower innovation inputs. Employing different samples, Palmer et al. (1995), Brännlund et al. (1995), and Barbers and McConnell (1990) prove that environmental regulation crowds out firm-financed spending on technological innovation and conclude that stringent environmental regulation would drive enterprises into a deteriorating situation. These scholars believe that environmental regulation is, under a static premise that technologies of enterprises, production procedure, and consumption demand remain unchanged, detrimental to technological innovation in enterprises.

On the other hand, some scholars adopt a dynamic perspective and explore environmental regulation’s compensation effects on corporate technological innovation in a bid to construe a win-win model between environmental protection and economic growth. Porter and van der Linde (1995) argue that “innovation offsets” brought by technological progress overpower environmental regulation costs as properly crafted environmental regulation can effectively induce regulated enterprises to innovate, hence the coinage of *Porter hypothesis*. Jaffe & Palmer (1997), Berman & Bui (2001), and Hamamoto (2006) examine the relation between environmental regulation and enterprise technological innovation based on samples from the U.S. and Japan, the results of which show the pressure put by environmental regulation can galvanize technological innovation among enterprises, testifying *Porter hypothesis*. Acemoglu et al. (2012) further divide production department into “clean” ones and “dirty” ones. Through construing a technological progress model, they systematically deduct the endogenous process of technological advancement and analyze impacts of environmental regulation on corporate technological innovation. Their numerical simulation results show that the combination of pollution tax and government financial support can facilitate innovation in clean technology and reduce emission while sustaining economic growth. In addition, some researchers believe that there exists a nonlinear relation between environmental regulation intensity and enterprise technological innovation, which presents a U-shape, namely a threshold value exists for environmental regulation to deliver effects (Dinda, 2004; Yin et al., 2015).

The second category of literature focuses on the impacts of government financial support on corporate technological innovation. Indeed, technological innovation is an investment which can benefit enterprises. However, risks of innovation, constrains on external financing, and overflow of innovation hinder enterprises from innovating. Government usually adopts measures like project funding, tax reduction or exemption, or R&D subsidy to motivate and support technological innovation in enterprises (Yu et al., 2016; Yuan & Zheng, 2017). Currently, the research results of government financial support’s impact on technological innovation by varied scholars are contentious. Researchers such as Clausen (2009), Bronzini & Piselli (2016), and Czarnitzki and Lopes-Bento (2014) hold that government financial support can lower enterprises’ R&D costs and risks, thus promoting their inputs in technological innovation. Of this, Bronzini & Piselli (2016) evaluate the impact of government financial support plan on patent applications in northern Italy through regression discontinuity and find that the subsidy plan boosts the possibility of patent applications significantly. In addition, Broekel et al. (2017) claim that government financial support impact region’s innovation growth when providing access to related variety and embedding regions into central positions in cross-regional knowledge network. Other researchers like Wallsten (2000), Görg & Strobl (2007) reveal that government financial support crowds out firm-financed R&D spending.

Based on the above research status, we find that it is generally accepted that environmental regulation and government financial support do affect enterprise technological innovation, whereas there is no reasonable and unified explanation of the influence directions and degrees of environmental regulation and government financial support on corporate technological innovation (Yu et al., 2018; Li et al., 2018; Zhang et al., 2019; Guo, 2019). Moreover, most studies focused on discussing the relationship between one specific factor (environmental regulation or government financial support) and enterprise technological innovation. However, there are few significant attempt to integrate environmental regulation, government financial support, and corporate technological innovation in a methodological framework (e.g., Féres & Reynaud, 2012; Van Rooij et al., 2013; Zhao and Sun, 2016; Guo, Qu & Tseng, 2017; and Guo et al., 2018). Zhao and Sun (2016) apply the panel regression models to investigate the effect of environmental regulation on corporation innovation and competitiveness using Chinese pollution-intensive corporation panel data for 2007 to 2012. Guo, Qu & Tseng (2017) use empirical data on 30 provincial administrative regions in China during 2011 to 2012 by employing structural equation modeling (SEM) approach to explore the effect of environmental regulation on technological innovation and green growth performance. These existing studies do lay the foundation and offer some inspiration for this paper, however, they emphasis on the direct effect of environmental regulation on enterprise technological innovation, ignoring exploring the moderating effects of environmental regulation on enterprise technological innovation at the regional level, which resulting from the interaction effects of environmental regulation and government financial support on corporate technological innovation. Thus, this paper develops an integrated model to investigate the relationship of environmental regulation, government financial support, and corporate technological innovation. Which aims to contribute to the nascent literature in technological innovation practices by incorporating government financial support into the model as an important factor and investigating the direct and moderating effects of environmental regulation intensity on enterprise technological innovation simultaneously.

Answer (4): Thanks a lot for your professional suggestions. Following your valuable suggestions, we have carefully read some new literature on the environmental regulation and added Professor T. Ramayah’s research works on green human resource management in recent years. We found that green human resource management approach is a promising method for people-management practice and environmental management. At the end of the article, we have mentioned green human resource management approach. Moreover, we have also added more recent works, such as: Yu, Wang, Xue, & Jin (2018), Li, Gu, Liu, & Li (2018), Zhang, Geng, Xu, et al. (2019), Guo (2019).

Answer (5): Thank you for your valuable questions and professional suggestions. Following your valuable suggestions, reference to the study of Yong, Yusliza, Ramayah, & Fawehinmi, O. (2019), we have re-edited the tables and figures, so as to all the tables and figures would be more professionally presented.

Answer (6): Thank you very much for your professional suggestions. Following your valuable suggestions, we have checked and found R2 of Table 2 is too high. Which is caused by “multicollinearity”, that is, the existence of such a high degree of correlation between supposedly independent variables being used to estimate a dependent variable that the contribution of each independent variable to variation in the dependent variable cannot be determined. This suggestion is very helpful for us to get more accurate empirical results. After analysis, we find that in the benchmark regression results, the explained variables “enterprise technological innovation” mainly uses the absolute value of R&D expenditure as an index, which has a certain economic scale effect and will be affected by the population and economy of the region. Further, the absolute value of R&D expenditure can’t well reflect the level of technological innovation of enterprises in a region. This may also be the reason why R2 is 90%+ in the empirical results. Therefore, we replace the absolute value of R&D expenditure with R&D input intensity (Ratio of R&D expenditure to GDP), and then make a regression analysis. The results are shown in Table 2. Table 2 shows that the size of R2 is 0.57+, which is relatively suitable.

**Table 2. Benchmark regression results for the whole sample.**

| **Variable** | **Explained Variable: ln*ETI*** | | |
| --- | --- | --- | --- |
|  | **Model (1)** | **Model (2)** | **Model (3)** |
| ln*ER* | -0.631^***^ | -0.632^***^ | -0.703^***^ |
|  | (-5.85) | (-5.86) | (-6.65) |
| ln*ER*^2^ | 0.056^***^ | 0.056^***^ | 0.016^*^ |
|  | (5.64) | (5.65) | (1.94) |
| ln*Gov_sub* |  | 0.017 | -0.166^***^ |
|  |  | (0.69) | (-3.29) |
| ln*ER*×ln*Gov_sub* |  |  | 0.045^***^ |
|  |  |  | (4.12) |
| ln*infra* | 0.244 | 0.240 | -0.198 |
|  | (0.51) | (0.45) | (-1.09) |
| ln*pgdp* | 0.637^**^ | 0.629^**^ | 0.572^**^ |
|  | (2.26) | (2.18) | (2.27) |
| ln*hcap* | 0.350 | 0.356^**^ | 0.432^**^ |
|  | (1.28) | (2.30) | (2.53) |
| *Tra* | -0.016 | -0.018 | -0.003 |
|  | (-0.18) | (-0.20) | (-0.03) |
| *fdi* | 0.061^*^ | 0.068^*^ | 0.006 |
|  | (1.77) | (1.85) | (0.07) |
| Constant | 0.829^**^ | 0.717^*^ | 1.955^*^ |
|  | (2.38) | (1.66) | (1.80) |
| Time fixed effect | Yes | Yes | Yes |
| Regional fixed effect | Yes | Yes | Yes |
| Observation | 270 | 270 | 270 |
| *R*^2^ | 0.574 | 0.573 | 0.601 |

*Notes*: All parameters are estimated based on the fixed effect model; the t statistical value is in parentheses under coefficients; *, **, and *** represent the significance at the 10%, 5%, and 1% levels, respectively.

Answer (7): Following your valuable suggestions, we have re-edited and added some discussion. First, to ensure the dependability of empirical results, we experiment several robustness checks, such as: replacing explained variables, replacing explanatory variables and utilize one-phase lagged core explanatory variables to estimate the above benchmark regression results. In more concrete terms, we replace the explained variable with successful patent applications (ln*ETI1*), which reflects corporate technological innovation outputs. Regression results are shown in Table 3. The regression results indicate that there is no explicit change on the numeric value of coefficient compared with the benchmark regression results, showcasing the consistency of conclusions. The impact of environmental regulation on technological innovation remains a U-shape relation. The sign and significance of the regression coefficient stay unchanged. With particular note, the coefficient of interaction term is also significantly positive, indicating that environmental regulation can enhance government financial support’s effect on enterprise technological innovation.

**Table 3. Results of robustness check replacing the explained variable with successful patent applications.**

| **Variable** | **Explained Variable：lnETI1** | | |
| --- | --- | --- | --- |
|  | **Model (4)** | **Model (5)** | **Model (6)** |
| ln*ER* | -0.453^***^ | -0.445^***^ | -0.506^***^ |
|  | (-3.32) | (-3.29) | (-3.75) |
| ln*ER*^2^ | 0.042^***^ | 0.041^***^ | 0.006^*^ |
|  | (3.35) | (3.30) | (1.83) |
| ln*Gov_sup* |  | -0.072 | -0.231^***^ |
|  |  | (-0.32) | (-3.59) |
| ln*ER*×ln*Gov_sup* |  |  | 0.039^***^ |
|  |  |  | (2.80) |
| ln*infra* | -0.015 | -0.036 | 0.000 |
|  | (-0.13) | (-0.29) | (0.00) |
| ln*pgdp* | 0.762^*^ | 0.695^*^ | 0.645^**^ |
|  | (1.88) | (1.93) | (2.37) |
| ln*hcap* | 0.584^*^ | 0.561 | 0.627^*^ |
|  | (1.69) | (1.63) | (1.85) |
| *Tra* | -0.056 | -0.047 | -0.033 |
|  | (-0.50) | (-0.42) | (-0.30) |
| *fdi* | 0.008 | 0.023^*^ | 0.077^**^ |
|  | (0.08) | (1.63) | (2.77) |
| Constant | -1.302 | -0.827 | 0.248^**^ |
|  | (-0.97) | (-0.61) | (2.58) |
| Time fixed effect | Yes | Yes | Yes |
| Regional fixed effect | Yes | Yes | Yes |
| Observation | 270 | 270 | 270 |
| *R*^2^ | 0.335 | 0.348 | 0.367 |

*Notes*: All parameters are estimated based on the fixed effect model; the t statistical value is in parentheses under coefficients; *, **, and *** represent the significance at the 10%, 5%, and 1% levels, respectively.

Moreover, we take pollution control costs per thousand industrial output as the proxy indicator for environmental regulation intensity and mark it as ln*ERs*. Of this, pollution control costs are the total operation costs of treatment facilities for industrial waste water and industrial waste gas. Estimation results are re-calculated and result is displayed in Table 4. The regression results indicate that there is no explicit change on the numeric value of coefficient compared with the benchmark regression results, showcasing the consistency of conclusions. The impact of environmental regulation on technological innovation remains a U-shape relation. The sign and significance of the regression coefficient stay unchanged. With particular note, the coefficient of interaction term is also significantly positive, indicating that environmental regulation can enhance government financial support’s effect on enterprise technological innovation.

**Table 4. Results of robustness check taking pollution control costs per thousand industrial output as the proxy indicator for environmental regulation.**

| **Variables** | **Explained Variable：*lnETI*** | | |
| --- | --- | --- | --- |
|  | **Model (1)** | **Model (2)** | **Model (3)** |
| ln*ER_s* | -0.207^**^ | -0.210^**^ | -0.396^**^ |
|  | (-2.08) | (-2.14) | (-2.32) |
| ln*ER_s* ^2^ | 0.047^**^ | 0.048^**^ | 0.048^**^ |
|  | (2.17) | (2.23) | (2.23) |
| ln*Gov_sup* |  | 0.076^*^ | 0.030 |
|  |  | (1.93) | (0.58) |
| ln*ER_s*×ln*Gov_sup* |  |  | 0.017^*^ |
|  |  |  | (1.96) |
| ln*infra* | -0.063 | -0.021 | -0.036 |
|  | (-0.51) | (-0.17) | (-0.29) |
| ln*pgdp* | 1.157^***^ | 1.065^***^ | 1.066^***^ |
|  | (4.91) | (4.48) | (4.50) |
| ln*hcap* | -0.743 | -0.627 | -0.694 |
|  | (-1.60) | (-1.36) | (-1.50) |
| *Tra* | 0.069 | 0.089 | 0.111 |
|  | (0.53) | (0.68) | (0.85) |
| *fdi* | 0.658^***^ | 0.638^***^ | 0.652^***^ |
|  | (3.17) | (3.10) | (3.18) |
| Constant | 3.720 | 3.492 | 4.157 |
|  | (1.34) | (1.28) | (1.50) |
| Time fixed effect | Yes | Yes | Yes |
| Regional fixed effect | Yes | Yes | Yes |
| Observation | 270 | 270 | 270 |
| *R*^2^ | 0.818 | 0.822 | 0.824 |

*Notes*: All parameters are estimated based on the fixed effect model; the t statistical value is in parentheses under coefficients; *, **, and *** represent the significance at the 10%, 5%, and 1% levels, respectively.

Besides, considering the potential endogeneity, the authors replace two key variables in major regression, namely environmental regulation intensity and government financial support, with their lagged variables. The quadratic and interaction term of the above two are also alternated with their lagged terms to mitigate biased error incurred by potential endogeneity. Table 5 shows the result. The regression results indicate that there is no explicit change on the numeric value of coefficient compared with the benchmark regression results, showcasing the consistency of conclusions. The impact of environmental regulation on technological innovation remains a U-shape relation. The sign and significance of the regression coefficient stay unchanged. With particular note, the coefficient of interaction term is also significantly positive, indicating that environmental regulation can enhance government financial support’s effect on enterprise technological innovation.

**Table 5. Results of robustness check with lagged explanatory variables.**

| **Variables** | **Explained Variable：ln*ETI*** | | |
| --- | --- | --- | --- |
|  | **Model (1)** | **Model (2)** | **Model (3)** |
| *lag_*ln*ER* | -0.558^***^ | -0.558^***^ | -0.710^***^ |
|  | (-4.79) | (-4.74) | (-5.73) |
| *lag_*ln*ER*^2^ | 0.051^***^ | 0.051^***^ | 0.021^*^ |
|  | (4.74) | (4.69) | (1.81) |
| *lag_*ln*Gov_sup* |  | -0.001 | -0.177^***^ |
|  |  | (-0.04) | (-2.99) |
| *lag_*ln*ER*×*lag_*ln*Gov_sup* |  |  | 0.042^***^ |
|  |  |  | (3.30) |
| ln*infra* | 0.180 | 0.181^*^ | 0.153 |
|  | (1.07) | (1.86) | (1.61) |
| ln*pgdp* | 0.768^**^ | 0.769^**^ | 0.626^*^ |
|  | (2.50) | (2.49) | (1.73) |
| ln*hcap* | 0.180^*^ | 0.179^*^ | 0.258^*^ |
|  | (1.65) | (1.64) | (1.95) |
| *Tra* | 0.129 | 0.129 | 0.134 |
|  | (1.37) | (1.37) | (1.46) |
| *fdi* | 0.069^**^ | 0.067^**^ | 0.104^*^ |
|  | (2.36) | (2.37) | (1.74) |
| Constant | 0.550^**^ | 0.561^**^ | 1.897^*^ |
|  | (2.48) | (2.28) | (1.87) |
| Time fixed effect | Yes | Yes | Yes |
| Regional fixed effect | Yes | Yes | Yes |
| Observation | 240 | 240 | 240 |
| *R*^2^ | 0.493 | 0.491 | 0.515 |

*Notes*: All parameters are estimated based on the fixed effect model; the t statistical value is in parentheses under coefficients; *, **, and *** represent the significance at the 10%, 5%, and 1% levels, respectively.

Second, we examine whether a regional heterogeneity exists due to regional differences in environmental regulation intensity and government financial support level. The vast land of China and differences in regional development give birth to regional heterogeneity of the direct and moderating effects of environmental regulation intensity on enterprise technological innovation. The samples are therefore grouped into eastern region samples, central region samples, and western region samples. Of this, eastern region includes *Beijing, Hebei, Shanghai, Zhejiang, Shandong, Tianjin, Liaoning, Jiangsu, Fujian, Guangdong*, and *Hainan*. Central region refers to *Inner Mongolia, Heilongjiang, Jiangxi, Hubei, Shanxi, Jilin, Anhui, Henan*, and *Hunan*. Western region includes *Guangxi, Guizhou, Shaanxi, Qinghai, Tibet, Sichuan, Yunnan, Gansu, Ningxia*, and *Chongqing*. The regression is conducted on the samples from three regions through fixed effects model. Table 7 shows the estimation results for eastern, central, and western China.

**Table 7. Regression results for eastern, central, and western China.**

| **Region** | **Model** | **ln*ER*** | **ln*ER*^2^** | **ln*Gov_sup*** | **ln*ER*×ln*Gov_sup*** | **Control Variable** | **Observation** | ***R*^2^** |
| --- | --- | --- | --- | --- | --- | --- | --- | --- |
| Eastern Region | Model (1) | -0.212^*^  (-1.88) | 0.017^*^  (1.83) |  |  | Yes | 99 | 0.65 |
|  | Model (2) | -0.218^*^  (-1.87) | 0.018^**^  (2.42) | -0.004  (-0.08) |  | Yes | 99 | 0.645 |
|  | Model (3) | -0.401  (-1.64) | 0.041^**^  (2.50) | -0.255^***^  (-2.72) | 0.072^***^  (3.16) | Yes | 99 | 0.685 |
| Central Region | Model (1) | -1.080^***^  (-5.08) | 0.097^***^  (4.96) |  |  | Yes | 81 | 0.844 |
|  | Model (2) | -1.126^***^  (-4.85) | 0.101^***^  (4.72) | -0.016  (-0.51) |  | Yes | 81 | 0.842 |
|  | Model (3) | -1.417^***^  (-4.86) | 0.088^***^  (3.90) | -0.213^*^  (-1.69) | 0.038  (1.61) | Yes | 81 | 0.846 |
| Western Region | Model (1) | -0.408^***^  (-2.97) | 0.038^**^  (2.41) |  |  | Yes | 90 | 0.52 |
|  | Model (2) | -0.406^***^  (-2.93) | 0.037^**^  (2.58) | -0.013  (-0.30) |  | Yes | 90 | 0.513 |
|  | Model (3) | -0.422^***^  (-3.08) | 0.014  (0.68) | -0.091^*^  (-1.73) | 0.024^*^  (1.70) | Yes | 90 | 0.527 |

*Notes*: All parameters are estimated based on the fixed effect model; the t statistical value is in parentheses under coefficients; *, **, and *** represent the significance at the 10%, 5%, and 1% levels, respectively.

Regression results reveal that estimated results of samples from three regions all showcase a nonlinear U-shape relation between environmental regulation intensity and corporate technological innovation. That is, enterprise technological innovation will fall at first and rise later as environmental regulation continues to be enhanced. The threshold value for reversing the trend is 6.235 in eastern region, 5.567 in central region, and 5.368 in western region. Apparently, the threshold value decreases from eastern to western region, demonstrating that environmental regulation intensity in eastern region to facilitate technological innovation of enterprises with an annual turnover of 20 million yuan is relatively higher while the environmental regulation intensity needed to enable technological innovation of enterprises with an annual turnover of over 20 million yuan in western region is the lowest. This may be attributable to the fact that enterprises in eastern region have a higher productivity, enjoy relatively loose financing environment, and are resourceful in various elements. They are more capable in handling the costs caused by strictly environmental regulation and requires a higher threshold value for environmental regulation to deliver its effects on enterprise technological innovation. Enterprises in central and western region face less favorable environment and their productivity is lower. They are more vulnerable to deal with the costs incurred by environmental regulation. The threshold value for environmental regulation to work on corporate technological innovation is accordingly lower.

Similar to the regression results, coefficient estimates of government financial support are not significant in all regions, meaning the direct effects of government financial support on technological innovation are not obvious in all regions. The comparison between the regression coefficient of the interaction term of environmental regulation and government financial support from different regions in model (3) shows that the regression coefficients of environmental regulation and government financial support in eastern and western region are positive. This proves that environmental regulation can intensify government financial support’s effects on technological innovation. Specifically, when ln*ER* in eastern region is higher than 3.542, the enabling effects of government financial support on technological innovation will be in place while ln*ER* in western region has to be higher than 3.792 for government financial support to deliver its effects. In addition, regression results of control variables are basically in line with previous results.

Answer (8): Following your valuable suggestions, we have added some more specific implications based on the quantitative analysis results. Findings in this paper carry broad implications. From the perspective of academic research, investigating the direct and moderating effects of environmental regulation intensity on enterprise technological innovation simultaneously and incorporating government financial support into the model as an important factor provide new insights into develop theories of environmental policy and management. For example, in the establishment and implement of China’s the environmental protection policy, which is more important: environmental regulation or government financial support? Whether there is interaction effects between environmental regulations and government financial support policies？How do government departments formulate environmental regulations and financial support policies to improve enterprise innovation? This study provides empirical evidence for better understand – and predict – the relationship of environmental regulation intensity, government financial support, and regional enterprise technological innovation to help guide the research in this filed.

From the perspective of the public policy-making, the research results of this paper show that there exists a U-shaped relation between environmental regulation intensity and technological innovation of enterprises which declines first and then climbs up. But government financial support does not significantly work on technological innovation directly, while environmental regulation drives this effect to be achieved, which highlights the complementarity between environmental regulation and government financial support. It suggests that enacting environmental regulation is the first step, but whether Chinese government can provide an effective incentive mechanism to firms to stimulate their technological innovation practices is another matter (Zhu et al., 2012, Guo, Qu, & Tseng, 2017). Hence, in order to change this relationship, it is necessary for Chinese government to adopt a hybrid approach combining government financial support and environmental regulation to motivate regional and enterprises’ technological innovation. Moreover, the government should further enhance environmental regulation intensity. On the one hand, it could force enterprises to apply technological innovation in pollution control and achieve pollution reduction and control in a high level. On the other hand, it could incentive enterprises to beef up innovation in production techniques and management methods (such as green human resource management), which is benefit for improving their productivity and international competitiveness (Yong, Yusliza, Ramayah, & Fawehinmi, 2019; Yong, Yusliza, Ramayah, & et al., 2019). But the government should also be alarmed that intensive environmental regulation may not be suitable to all region. Instead, the government ought to, based on the actual condition of specific region and sector, implement targeted and differentiated environmental regulation and make timely adjustments, rather than insist on a fixed standard, toward a proper range so as to keep the enabling effects of environmental regulation working (Porter, 1995).

From the perspective of the policy management and operational level, the government should identify the appropriate range for subsidy, but it should be considered synthetically from the whole angle, and adjusted dynamically at any time. Due to the positive externality of innovation and the crowding-out of R&D expense by environmental regulation, lack of funding in technological innovation is natural. In order to promote the interaction effects of environmental regulation and government financial support on enterprise technological innovation, the government should complement environmental regulation through reasonable funding and policy support. But this does not mean that the government should provide as much subsidy as possible. There exists a threshold value and proper range for environmental regulation to work on technological innovation effectively. When environmental regulation passes the inflection point and begins to enable technological innovation, government subsidy focusing on technological innovation can be cut down or even lifted.

Answer (9): Following your valuable suggestions, we have added some limitations and suggestions for future research. (1) Limitations in the measurement of variables: Existing studies on the relationship between environmental regulation and enterprise technological innovation in China have failed to effectively control for the impact of government financial support because of data limitations. For this research, a government financial support variable, based on the government-financed fund in R&D expense data, is developed and incorporated into the model, and while this variable is relatively consistent with the overall government financial support, there are still cannot distinguish whether it is for environmental-friendly technologies or not. In follow-up studies, additional data on government funding for green technologies need to be compiled, and combined with green innovation for further analysis. (2) Environmental regulations can be further subdivided: For example, it is possible to decompose environmental regulations into different sub-components based on type of regulation, such as the formal and informal environmental regulation. Further, it is also possible that testing the effect of environmental regulations against a wide range of different measures of technological innovation. However, due to manufacturing industry and regional panel data limitations, we are not yet able to examine this issue more extensively. The resulting problems need to be explored and new solutions developed in follow-up research. Therefore, in the latter study, we will try to use micro-enterprise level data to study the interaction effects of environmental regulation and government funding for green technologies on green innovation behavior of enterprises, and consider that in the different types of environmental regulation.

Response to Reviewer 2 Comments:

We are very grateful to you for your comments and suggestions. Thanks a lot for your encouragement. We greatly appreciate your time and very professional and helpful comments. We have carefully read some new literature on the environmental regulation and done more work to link the development skeleton of the literature clearly. We also rewrote some of the abstract, literature review, results and discussion. Moreover, we added a graphical abstract to the manuscript. Furthermore, at the end of the article, based on the quantitative analysis results, we added some more specific implications and some limitations and suggestions for future research. More details can be found in the revised portions, which are shown in red in the manuscript. Our responses are provided as follows.

Answer (1): Thank you for your valuable questions and professional suggestions. Following your valuable suggestions, we have carefully read some new literature on the environmental regulation and done more work to link the development skeleton of the literature clearly. Moreover, we have also added more recent works, such as: Yu, Wang, Xue, & Jin (2018), Li, Gu, Liu, & Li (2018), Zhang, Geng, Xu, et al. (2019), Guo (2019). Finally, we have re-edited literature review so as to clearly present the gap within the current literature. The literature review is shown as follows:

Our article relates to two main strands of the literature. The first category of literature concentrates on environmental regulation’s effects on enterprise technological innovation, and it was first mentioned by Magat (1978) who believes that innovation is the key to dissolve the contradiction between environmental protection and economic development. But opinions on the relationship between these two diverge. On the one hand, traditional neoclassical theory holds that environmental regulation can improve social benefits on the whole but will inevitably increase the costs of enterprises, lowering their capability in technological innovation. This constitutes the “compliance costs” entailed by environmental regulation. Gray (1987) reveals, based on the data from American manufacturing and power industry, the negative effects of environmental regulation on innovation level and economic growth as environmental regulation would increase enterprises’ costs in emission reduction and lower innovation inputs. Employing different samples, Palmer et al. (1995), Brännlund et al. (1995), and Barbers and McConnell (1990) prove that environmental regulation crowds out firm-financed spending on technological innovation and conclude that stringent environmental regulation would drive enterprises into a deteriorating situation. These scholars believe that environmental regulation is, under a static premise that technologies of enterprises, production procedure, and consumption demand remain unchanged, detrimental to technological innovation in enterprises.

On the other hand, some scholars adopt a dynamic perspective and explore environmental regulation’s compensation effects on corporate technological innovation in a bid to construe a win-win model between environmental protection and economic growth. Porter and van der Linde (1995) argue that “innovation offsets” brought by technological progress overpower environmental regulation costs as properly crafted environmental regulation can effectively induce regulated enterprises to innovate, hence the coinage of *Porter hypothesis*. Jaffe & Palmer (1997), Berman & Bui (2001), and Hamamoto (2006) examine the relation between environmental regulation and enterprise technological innovation based on samples from the U.S. and Japan, the results of which show the pressure put by environmental regulation can galvanize technological innovation among enterprises, testifying *Porter hypothesis*. Acemoglu et al. (2012) further divide production department into “clean” ones and “dirty” ones. Through construing a technological progress model, they systematically deduct the endogenous process of technological advancement and analyze impacts of environmental regulation on corporate technological innovation. Their numerical simulation results show that the combination of pollution tax and government financial support can facilitate innovation in clean technology and reduce emission while sustaining economic growth. In addition, some researchers believe that there exists a nonlinear relation between environmental regulation intensity and enterprise technological innovation, which presents a U-shape, namely a threshold value exists for environmental regulation to deliver effects (Dinda, 2004; Yin et al., 2015).

The second category of literature focuses on the impacts of government financial support on corporate technological innovation. Indeed, technological innovation is an investment which can benefit enterprises. However, risks of innovation, constrains on external financing, and overflow of innovation hinder enterprises from innovating. Government usually adopts measures like project funding, tax reduction or exemption, or R&D subsidy to motivate and support technological innovation in enterprises (Yu et al., 2016; Yuan & Zheng, 2017). Currently, the research results of government financial support’s impact on technological innovation by varied scholars are contentious. Researchers such as Clausen (2009), Bronzini & Piselli (2016), and Czarnitzki and Lopes-Bento (2014) hold that government financial support can lower enterprises’ R&D costs and risks, thus promoting their inputs in technological innovation. Of this, Bronzini & Piselli (2016) evaluate the impact of government financial support plan on patent applications in northern Italy through regression discontinuity and find that the subsidy plan boosts the possibility of patent applications significantly. In addition, Broekel et al. (2017) claim that government financial support impact region’s innovation growth when providing access to related variety and embedding regions into central positions in cross-regional knowledge network. Other researchers like Wallsten (2000), Görg & Strobl (2007) reveal that government financial support crowds out firm-financed R&D spending.

Based on the above research status, we find that it is generally accepted that environmental regulation and government financial support do affect enterprise technological innovation, whereas there is no reasonable and unified explanation of the influence directions and degrees of environmental regulation and government financial support on corporate technological innovation (Yu et al., 2018; Li et al., 2018; Zhang et al., 2019; Guo, 2019). Moreover, most studies focused on discussing the relationship between one specific factor (environmental regulation or government financial support) and enterprise technological innovation. However, there are few significant attempt to integrate environmental regulation, government financial support, and corporate technological innovation in a methodological framework (e.g., Féres & Reynaud, 2012; Van Rooij et al., 2013; Zhao and Sun, 2016; Guo, Qu & Tseng, 2017; and Guo et al., 2018). Zhao and Sun (2016) apply the panel regression models to investigate the effect of environmental regulation on corporation innovation and competitiveness using Chinese pollution-intensive corporation panel data for 2007 to 2012. Guo, Qu & Tseng (2017) use empirical data on 30 provincial administrative regions in China during 2011 to 2012 by employing structural equation modeling (SEM) approach to explore the effect of environmental regulation on technological innovation and green growth performance. These existing studies do lay the foundation and offer some inspiration for this paper, however, they emphasis on the direct effect of environmental regulation on enterprise technological innovation, ignoring exploring the moderating effects of environmental regulation on enterprise technological innovation at the regional level, which resulting from the interaction effects of environmental regulation and government financial support on corporate technological innovation. Thus, this paper develops an integrated model to investigate the relationship of environmental regulation, government financial support, and corporate technological innovation. Which aims to contribute to the nascent literature in technological innovation practices by incorporating government financial support into the model as an important factor and investigating the direct and moderating effects of environmental regulation intensity on enterprise technological innovation simultaneously.

The graphical abstract is shown as below:

Environmental regulation intensity

Environmental regulation intensity

Direct effects
(U-shaped relation)

The linear moderating effects

(significantly)

Government financial support

Environmental regulation intensity

Enterprise technological innovation

Environmental regulation intensity

Direct effects
(not significantly)

)

Answer (2): Thank you for your valuable questions and professional suggestions. Following your valuable suggestions, we have added the difference of the research results and some more specific implications.

China currently faces the challenge imposed by sustaining economic growth and mitigating environmental degradation. The key to resolving the dilemma lies in the upgrading of technological innovation intensity. According to Porter hypothesis, properly designed environmental regulation can trigger “innovation offsets”，compensate “compliance cost”, and boost corporate productivity and competitiveness. Moreover, government R&D subsidy, directly or indirectly, work on technological innovation when there are environmental regulation constrains. Although there are some studies on the impacts of environmental regulation and government R&D subsidy on the achievement of green growth, they are limited in quantity. Moreover, most studies focused on discussing the relationship between one specific factor (environmental regulation or government financial support) and enterprise technological innovation. However, there are few significant attempt to integrate environmental regulation, government financial support, and corporate technological innovation in a methodological framework (e.g., Féres & Reynaud, 2012; Van Rooij et al., 2013; Zhao and Sun, 2016; Guo, Qu & Tseng, 2017; and Guo et al., 2018). Accordingly, this paper develops an integrated model to investigate the relationship of environmental regulation, government financial support, and corporate technological innovation. Employing the data of the industrial enterprises with an annual turnover of over 20 million yuan from 30 Chinese provinces or municipalities between 2008 and 2016, this paper applies the fixed effect regression model to reveal the direct and moderating effects of environmental regulation intensity on enterprise technological innovation, and the impacts of government financial support on corporate technological innovation. Results show that: (1) there exists a U-shaped relation between environmental regulation intensity and technological innovation of enterprises which declines first and then climbs up, and China is still at the stage of inhibition before the “inflection point”. (2) government financial support does not significantly work on technological innovation directly, but environmental regulation drives this effect to be achieved; when the value of ln*ER* is higher than 3.69, government financial support can significantly facilitate corporate technological innovation. (3) the comparison between regional samples reveals that heterogeneity exists in the influence of environmental regulation intensity and government financial support on corporate technological innovation. The threshold value of enabling effects of environmental regulation in eastern region is higher than that of the central and western region. These results remain consistent after we experiment several robustness checks.

Findings in this paper carry broad implications. From the perspective of academic research, investigating the direct and moderating effects of environmental regulation intensity on enterprise technological innovation simultaneously and incorporating government financial support into the model as an important factor provide new insights into develop theories of environmental policy and management. For example, in the establishment and implement of China’s the environmental protection policy, which is more important: environmental regulation or government financial support? Whether there is interaction effects between environmental regulations and government financial support policies？How do government departments formulate environmental regulations and financial support policies to improve enterprise innovation? This study provides empirical evidence for better understand – and predict – the relationship of environmental regulation intensity, government financial support, and regional enterprise technological innovation to help guide the research in this filed.

From the perspective of the public policy-making, the research results of this paper show that there exists a U-shaped relation between environmental regulation intensity and technological innovation of enterprises which declines first and then climbs up. But government financial support does not significantly work on technological innovation directly, while environmental regulation drives this effect to be achieved, which highlights the complementarity between environmental regulation and government financial support. It suggests that enacting environmental regulation is the first step, but whether Chinese government can provide an effective incentive mechanism to firms to stimulate their technological innovation practices is another matter (Zhu et al., 2012, Guo, Qu, & Tseng, 2017). Hence, in order to change this relationship, it is necessary for Chinese government to adopt a hybrid approach combining government financial support and environmental regulation to motivate regional and enterprises’ technological innovation. Moreover, the government should further enhance environmental regulation intensity. On the one hand, it could force enterprises to apply technological innovation in pollution control and achieve pollution reduction and control in a high level. On the other hand, it could incentive enterprises to beef up innovation in production techniques and management methods (such as green human resource management), which is benefit for improving their productivity and international competitiveness (Yong, Yusliza, Ramayah, & Fawehinmi, 2019; Yong, Yusliza, Ramayah, & et al., 2019). But the government should also be alarmed that intensive environmental regulation may not be suitable to all region. Instead, the government ought to, based on the actual condition of specific region and sector, implement targeted and differentiated environmental regulation and make timely adjustments, rather than insist on a fixed standard, toward a proper range so as to keep the enabling effects of environmental regulation working (Porter, 1995).

From the perspective of the policy management and operational level, the government should identify the appropriate range for subsidy, but it should be considered synthetically from the whole angle, and adjusted dynamically at any time. Due to the positive externality of innovation and the crowding-out of R&D expense by environmental regulation, lack of funding in technological innovation is natural. In order to promote the interaction effects of environmental regulation and government financial support on enterprise technological innovation, the government should complement environmental regulation through reasonable funding and policy support. But this does not mean that the government should provide as much subsidy as possible. There exists a threshold value and proper range for environmental regulation to work on technological innovation effectively. When environmental regulation passes the inflection point and begins to enable technological innovation, government subsidy focusing on technological innovation can be cut down or even lifted.

Moreover, we have added some limitations and suggestions for future research. (1) Limitations in the measurement of variables: Existing studies on the relationship between environmental regulation and enterprise technological innovation in China have failed to effectively control for the impact of government financial support because of data limitations. For this research, a government financial support variable, based on the government-financed fund in R&D expense data, is developed and incorporated into the model, and while this variable is relatively consistent with the overall government financial support, there are still cannot distinguish whether it is for environmental-friendly technologies or not. In follow-up studies, additional data on government funding for green technologies need to be compiled, and combined with green innovation for further analysis. (2) Environmental regulations can be further subdivided: For example, it is possible to decompose environmental regulations into different sub-components based on type of regulation, such as the formal and informal environmental regulation. Further, it is also possible that testing the effect of environmental regulations against a wide range of different measures of technological innovation. However, due to manufacturing industry and regional panel data limitations, we are not yet able to examine this issue more extensively. The resulting problems need to be explored and new solutions developed in follow-up research. Therefore, in the latter study, we will try to use micro-enterprise level data to study the interaction effects of environmental regulation and government funding for green technologies on green innovation behavior of enterprises, and consider that in the different types of environmental regulation.

Response to Reviewer 3 Comments:

We are very grateful to you for your comments and suggestions. Thanks a lot for your encouragement. We greatly appreciate your time and very professional and helpful comments. we have applied the micro-enterprise level data to study the interaction effects of environmental regulation and government funding for green technologies on green innovation behavior of enterprises. Moreover, we have carefully read some new literature on the environmental regulation and done more work to link the development skeleton of the literature clearly. We also rewrote some of the abstract, literature review, results and discussion. Furthermore, at the end of the article, based on the quantitative analysis results, we added some more specific implications and some limitations and suggestions for future research. More details can be found in the revised portions, which are shown in red in the manuscript. Our responses are provided as follows.

Answer (1): Thank you for your valuable questions and professional suggestions. As you said, for this research, a government financial support variable, based on the government-financed fund in R&D expense data, is developed and incorporated into the model, and while this variable is relatively consistent with the overall government financial support, there are still cannot distinguish whether it is for environmental-friendly technologies or not. In follow-up studies, additional data on government funding for green technologies need to be compiled, and combined with green innovation for further analysis. However, due to data constraints and the setting of research topics, this paper cannot directly explore the interaction effects of environmental regulation and government funding for green technologies on green innovation behavior of enterprises. This paper is focused on developing an integrated model to investigate the relationship of environmental regulation, government financial support, and corporate technological innovation at the regional level. But your idea is very valuable. This is our further research direction. We try our best to collect the micro-enterprise level data. Fortunately, we find that there are government environmental protection subsidies needed in the data of Listed Companies in China. On the network of the *State Intellectual Property Office of China*, we collect enterprise green patent data by inputting IPC classification number for retrieval.

**1. Sample selection and data sources**

In order to investigate the interaction effects of environmental regulation and government funding for green technologies on green innovation of enterprises, we mainly select the data of listed Companies in the A-share market from 2008 to 2017 as the research sample. Before data analysis, referring to the existing literature (Gu Xiamen et al., 2018; Wang Zheng et al., 2018), we processed the data as follows: (1) excluding samples of listed companies that were specially processed during the observation period (ST, * ST, etc.); (2) excluding samples of financial and insurance companies; (3) excluding those whose asset-liability ratio is greater than 1. (4) Exclude the samples with serious missing data. In order to avoid the influence of extreme values on the empirical results, the main continuous variables are processed by Winsorize at the 1% and 99% levels. The basic information and financial data of R&D investment, government environmental subsidies and other listed companies in this paper are from CSMAR database and WIND database. The data collected in this paper are from the network of the State Intellectual Property Office. The data sources of other variables in this paper are consistent with the text.

**2. Selection of Variables**

the interaction effects of environmental regulation and government funding for green technologies on green innovation of enterprises, this paper mainly regards technological innovation of enterprises as the explained variable, environmental regulation and government environmental protection subsidy as the core explanatory variable. In addition, this paper also considers the impact of other factors on technological innovation of enterprises, and then controls the variables such as enterprise characteristics and regional characteristics. Specific variables are defined as follows:

*1. Explained variable*

Enterprise green technological innovation is the explained variable in this paper. This paper uses two criteria to measure the level of technological innovation of enterprises. One is from the perspective of innovation input, and the other is from the perspective of innovation output. It chooses R&D input intensity (*R&D_int*) to measure the level of technological innovation input of enterprises. The specific calculation method of this index is the percentage of R&D expenditure in business income. The other is from the perspective of innovation output. Number of green patent applications (*Green_patent*) is used to measure the innovation output of enterprises. Referring to Levine et al. (2015) method, the number of patent applications plus 1 is used as agent index. This paper defines patent output according to application time rather than granting time, because patent application time can more accurately reflect the actual time of enterprise innovation output (Cornaggia et al., 2015).

*2. Explanatory variables*

Environmental regulation is the key explanatory variable in this paper. The measurement method of environmental regulation in this part is consistent with the original text, and it will not be repeated here.

Government funding for green technologies is also the key explanatory variable of this paper. Government support for enterprises mainly includes direct financial support, tax incentives, government procurement and other ways. Government subsidies are mostly in the form of cash subsidies, tax returns and incentives. This part mainly studies government funding for green technologies, so when calculating the government subsidy of each enterprise in that year, only the subsidy related to environmental protection is retained (such as subsidy for improving atmospheric environmental quality, support fund for meeting environmental protection standards, subsidy for environmental protection and energy saving projects, local subsidy for reducing emissions of enterprises, etc.) and the government is measured by it. The government funding for green technologies (ln*Gov_ensub*) has been processed logarithmically.

*3. Other control variables*

According to the existing research, this part mainly controls the variables such as enterprise characteristics and regional characteristics that affect enterprise technological innovation. Variables at the enterprise level include the ownership nature of enterprises (*SOE*), size of enterprises (*Size*), firm age (*Age*), asset-liability ratio (*Lever*), the return on assets (*Roa*), cash flow ratio (*CF*), Shareholding proportion of the largest shareholder (*Lash*) and Percentage of fixed assets (*Fix_ratio*). Variables at the regional level variables include: infrastructure (*lninfra*), per capita GDP (*lnpgdp*), level of opening up (*Tra*) and level of human capital (*lnhcap*). In addition, this paper also controls the year dummy variable, provincial dummy variable, industry dummy variable and enterprise dummy variable.

**3. Analysis of empirical results**

This paper uses the panel data of of listed companies in the A-share market from 2008 to 2017 to explore the interaction effects of environmental regulation and government funding for green technologies on green innovation of enterprises. The results of *Hausman* test reveal that fixed effects model are better than random effects model. This is because the samples of the research are regions with their own characteristics, which would affect the regression analyses results. To this end, the authors employ fixed effects model, controlling provincial fixed effects, industry fixed effects, enterprise fixed effects, and yearly fixed effects, to unravel the direct and moderating effect of environmental regulation intensity on green innovation of enterprises, as well as the effect of government funding for green technologies on green innovation of enterprises. The explained variables in model (1) and (2) of Table 1 are R&D input intensity (*R&D_int*), and the explained variables in model (3) and (4) are number of green patent applications (*Green_patent*). Regression results are shown in Table 1. From regression results, the authors discover that coefficient estimates of environmental regulation intensity are all negative in model (1) and (3) while coefficient estimates of the quadratic term of environmental regulation intensity are all positive and significant at the 5% level. Which shows that the relationship of environmental regulation and green innovation of enterprises emerges as a U-shape. That is to say, the green technological innovation of enterprises will first decrease and then increase with the enhancement of environmental regulation. Which is consistent with the conclusions of the original text. Moreover, the threshold value is within the value range of ln*ER*, signifying that China is still at the stage of inhibition before the “inflection point” and environmental regulation intensity should be enhanced.

**Table 1. The interaction effects of environmental regulation and government funding for green technologies on green innovation of enterprises.**

| **Variables** | **Model (1)** | **Model (2)** | **Model (3)** | **Model (4)** |
| --- | --- | --- | --- | --- |
|  | ***R&D_int*** | ***R&D_int*** | ***green_patent*** | ***green_patent*** |
| ln*ER* | -0.467 | -0.648 | -0.035 | 0.050 |
|  | (0.481) | (0.507) | (0.174) | (0.177) |
| ln*ER*^2^ | 0.538^**^ | 0.018 | 0.215^***^ | 0.247^***^ |
|  | (0.254) | (0.039) | (0.080) | (0.085) |
| *lnER*×*lnGov_ensub* |  | 0.053^**^ |  | 0.015^***^ |
|  |  | (0.026) |  | (0.003) |
| *lnGov_ensub* |  | -0.241 |  | -0.048^**^ |
|  |  | (0.155) |  | (0.019) |
| *SOE* | -0.265 | -0.288 | -0.115 | -0.085 |
|  | (0.259) | (0.264) | (0.092) | (0.097) |
| *CF* | -0.193 | -0.267 | -0.085 | -0.046 |
|  | (0.301) | (0.310) | (0.088) | (0.099) |
| *Size* | -0.142^*^ | -0.200^**^ | 0.300^***^ | 0.311^***^ |
|  | (0.079) | (0.081) | (0.024) | (0.027) |
| *Lever* | -2.046^***^ | -1.962^***^ | 0.005 | -0.343^***^ |
|  | (0.266) | (0.259) | (0.016) | (0.098) |
| *ROA* | -3.370^***^ | -3.292^***^ | -0.198** | -0.341^*^ |
|  | (0.649) | (0.653) | (0.085) | (0.178) |
| *Fix_ratio* | 0.247 | 0.162 | 0.013 | 0.051 |
|  | (0.302) | (0.309) | (0.110) | (0.119) |
| *Age* | -0.861^***^ | -0.777^***^ | 0.372^***^ | 0.313^***^ |
|  | (0.237) | (0.226) | (0.086) | (0.088) |
| *Large share* | -0.094 | -0.180 | -0.721^***^ | -0.706^***^ |
|  | (0.383) | (0.385) | (0.169) | (0.179) |
| *lninfra* | 0.035 | 0.103 | -0.007 | 0.043 |
|  | (0.144) | (0.178) | (0.049) | (0.060) |
| *lnpgdp* | 2.007^***^ | 2.713^***^ | 0.721^***^ | 0.540^***^ |
|  | (0.486) | (0.357) | (0.170) | (0.181) |
| *lnhcap* | 0.015 | -0.086 | -0.041 | -0.068 |
|  | (0.195) | (0.207) | (0.057) | (0.060) |
| *Tra* | -0.427 | -0.803^**^ | 0.119 | 0.370^**^ |
|  | (0.379) | (0.402) | (0.137) | (0.147) |
| Constant | -14.274^**^ | -20.108^***^ | -13.332^***^ | -10.319^***^ |
|  | (0.556) | (0.664) | (0.188) | (0.355) |
| Time fixed effect | Yes | Yes | Yes | Yes |
| Regional fixed effect | Yes | Yes | Yes | Yes |
| Industry fixed effect | Yes | Yes | Yes | Yes |
| Enterprise fixed effect | Yes | Yes | Yes | Yes |
| Observation | 13126 | 12823 | 18406 | 17286 |
| *R*^2^ | 0.161 | 0.161 | 0.217 | 0.231 |

*Notes*: All parameters are estimated based on the fixed effect model; the t statistical value is in parentheses under coefficients; *, **, and *** represent the significance at the 10%, 5%, and 1% levels, respectively.

The interaction term of the government funding for green technologies and environmental regulation intensity (ln*ER*×ln*Gov_sup*) are also integrated into model (2) and (4). The result shows that the coefficient estimates of government funding for green technologies is positive, but it isn’t significant, demonstrating that government funding for green technologies does not significantly work on green technological innovation of enterprises directly. The coefficient estimates of interaction term (ln*ER*×ln*Gov_sup*) is positive and significant at the 5% level. Which proves that environmental regulation can enhance government funding for green technologies’ effect on green technological innovation of enterprises. This is also consistent with the conclusions of the original text. Therefore, we the empirical results based on the data from the micro-level of the enterprise are consistent consistent with the conclusions of the original text.

Finally, I must admit that it’s a valuable idea for exploring the interaction effects of environmental regulation and government funding for green technologies on green innovation of enterprises. Based on this, we can more accurately identify the role of environmental regulation and environmental R&D subsidies in promoting green technology. In the latter study, we will decompose environmental regulations into different sub-components based on type of regulation, such as the formal and informal environmental regulation. Moreover, it is also possible that testing the effect of environmental regulations against a wide range of different measures of technological innovation. I hope we can have the opportunity to further consult and learn from you.

Answer (2): Thank you for your valuable questions and professional suggestions. Following your valuable suggestions, we have carefully read some new literature on the environmental regulation and done more work to link the development skeleton of the literature clearly. Moreover, we have also added more recent works of Chinese peer-review journals, such as: Yu, Wang, Xue, & Jin (2018), Li, Gu, Liu, & Li (2018), Zhang, Geng, Xu, et al. (2019), Guo (2019). Finally, we have re-edited literature review so as to clearly present the gap within the current literature. The literature review is shown as follows:

Our article relates to two main strands of the literature. The first category of literature concentrates on environmental regulation’s effects on enterprise technological innovation, and it was first mentioned by Magat (1978) who believes that innovation is the key to dissolve the contradiction between environmental protection and economic development. But opinions on the relationship between these two diverge. On the one hand, traditional neoclassical theory holds that environmental regulation can improve social benefits on the whole but will inevitably increase the costs of enterprises, lowering their capability in technological innovation. This constitutes the “compliance costs” entailed by environmental regulation. Gray (1987) reveals, based on the data from American manufacturing and power industry, the negative effects of environmental regulation on innovation level and economic growth as environmental regulation would increase enterprises’ costs in emission reduction and lower innovation inputs. Employing different samples, Palmer et al. (1995), Brännlund et al. (1995), and Barbers and McConnell (1990) prove that environmental regulation crowds out firm-financed spending on technological innovation and conclude that stringent environmental regulation would drive enterprises into a deteriorating situation. These scholars believe that environmental regulation is, under a static premise that technologies of enterprises, production procedure, and consumption demand remain unchanged, detrimental to technological innovation in enterprises.

On the other hand, some scholars adopt a dynamic perspective and explore environmental regulation’s compensation effects on corporate technological innovation in a bid to construe a win-win model between environmental protection and economic growth. Porter and van der Linde (1995) argue that “innovation offsets” brought by technological progress overpower environmental regulation costs as properly crafted environmental regulation can effectively induce regulated enterprises to innovate, hence the coinage of *Porter hypothesis*. Jaffe & Palmer (1997), Berman & Bui (2001), and Hamamoto (2006) examine the relation between environmental regulation and enterprise technological innovation based on samples from the U.S. and Japan, the results of which show the pressure put by environmental regulation can galvanize technological innovation among enterprises, testifying *Porter hypothesis*. Acemoglu et al. (2012) further divide production department into “clean” ones and “dirty” ones. Through construing a technological progress model, they systematically deduct the endogenous process of technological advancement and analyze impacts of environmental regulation on corporate technological innovation. Their numerical simulation results show that the combination of pollution tax and government financial support can facilitate innovation in clean technology and reduce emission while sustaining economic growth. In addition, some researchers believe that there exists a nonlinear relation between environmental regulation intensity and enterprise technological innovation, which presents a U-shape, namely a threshold value exists for environmental regulation to deliver effects (Dinda, 2004; Yin et al., 2015).

The second category of literature focuses on the impacts of government financial support on corporate technological innovation. Indeed, technological innovation is an investment which can benefit enterprises. However, risks of innovation, constrains on external financing, and overflow of innovation hinder enterprises from innovating. Government usually adopts measures like project funding, tax reduction or exemption, or R&D subsidy to motivate and support technological innovation in enterprises (Yu et al., 2016; Yuan & Zheng, 2017). Currently, the research results of government financial support’s impact on technological innovation by varied scholars are contentious. Researchers such as Clausen (2009), Bronzini & Piselli (2016), and Czarnitzki and Lopes-Bento (2014) hold that government financial support can lower enterprises’ R&D costs and risks, thus promoting their inputs in technological innovation. Of this, Bronzini & Piselli (2016) evaluate the impact of government financial support plan on patent applications in northern Italy through regression discontinuity and find that the subsidy plan boosts the possibility of patent applications significantly. In addition, Broekel et al. (2017) claim that government financial support impact region’s innovation growth when providing access to related variety and embedding regions into central positions in cross-regional knowledge network. Other researchers like Wallsten (2000), Görg & Strobl (2007) reveal that government financial support crowds out firm-financed R&D spending.

Based on the above research status, we find that it is generally accepted that environmental regulation and government financial support do affect enterprise technological innovation, whereas there is no reasonable and unified explanation of the influence directions and degrees of environmental regulation and government financial support on corporate technological innovation (Yu et al., 2018; Li et al., 2018; Zhang et al., 2019; Guo, 2019). Moreover, most studies focused on discussing the relationship between one specific factor (environmental regulation or government financial support) and enterprise technological innovation. However, there are few significant attempt to integrate environmental regulation, government financial support, and corporate technological innovation in a methodological framework (e.g., Féres & Reynaud, 2012; Van Rooij et al., 2013; Zhao and Sun, 2016; Guo, Qu & Tseng, 2017; and Guo et al., 2018). Zhao and Sun (2016) apply the panel regression models to investigate the effect of environmental regulation on corporation innovation and competitiveness using Chinese pollution-intensive corporation panel data for 2007 to 2012. Guo, Qu & Tseng (2017) use empirical data on 30 provincial administrative regions in China during 2011 to 2012 by employing structural equation modeling (SEM) approach to explore the effect of environmental regulation on technological innovation and green growth performance. These existing studies do lay the foundation and offer some inspiration for this paper, however, they emphasis on the direct effect of environmental regulation on enterprise technological innovation, ignoring exploring the moderating effects of environmental regulation on enterprise technological innovation at the regional level, which resulting from the interaction effects of environmental regulation and government financial support on corporate technological innovation. Thus, this paper develops an integrated model to investigate the relationship of environmental regulation, government financial support, and corporate technological innovation. Which aims to contribute to the nascent literature in technological innovation practices by incorporating government financial support into the model as an important factor and investigating the direct and moderating effects of environmental regulation intensity on enterprise technological innovation simultaneously.

The graphical abstract is shown as below:

Environmental regulation intensity

Environmental regulation intensity

Direct effects
(U-shaped relation)

The linear moderating effects

(significantly)

Government financial support

Environmental regulation intensity

Enterprise technological innovation

Environmental regulation intensity

Direct effects
(not significantly)

)

Answer (3): Thank you for your valuable questions and professional suggestions. This study covers three major areas: policy, economy and management. It should be regarded as an interdisciplinary study. Moreover, following your valuable suggestions, we have added the further analysis of the research results and some more specific implications.

China currently faces the challenge imposed by sustaining economic growth and mitigating environmental degradation. The key to resolving the dilemma lies in the upgrading of technological innovation intensity. According to Porter hypothesis, properly designed environmental regulation can trigger “innovation offsets”，compensate “compliance cost”, and boost corporate productivity and competitiveness. Moreover, government R&D subsidy, directly or indirectly, work on technological innovation when there are environmental regulation constrains. Although there are some studies on the impacts of environmental regulation and government R&D subsidy on the achievement of green growth, they are limited in quantity. Moreover, most studies focused on discussing the relationship between one specific factor (environmental regulation or government financial support) and enterprise technological innovation. However, there are few significant attempt to integrate environmental regulation, government financial support, and corporate technological innovation in a methodological framework (e.g., Féres & Reynaud, 2012; Van Rooij et al., 2013; Zhao and Sun, 2016; Guo, Qu & Tseng, 2017; and Guo et al., 2018). Accordingly, this paper develops an integrated model to investigate the relationship of environmental regulation, government financial support, and corporate technological innovation. Employing the data of the industrial enterprises with an annual turnover of over 20 million yuan from 30 Chinese provinces or municipalities between 2008 and 2016, this paper applies the fixed effect regression model to reveal the direct and moderating effects of environmental regulation intensity on enterprise technological innovation, and the impacts of government financial support on corporate technological innovation. Results show that: (1) there exists a U-shaped relation between environmental regulation intensity and technological innovation of enterprises which declines first and then climbs up, and China is still at the stage of inhibition before the “inflection point”. (2) government financial support does not significantly work on technological innovation directly, but environmental regulation drives this effect to be achieved; when the value of ln*ER* is higher than 3.69, government financial support can significantly facilitate corporate technological innovation. (3) the comparison between regional samples reveals that heterogeneity exists in the influence of environmental regulation intensity and government financial support on corporate technological innovation. The threshold value of enabling effects of environmental regulation in eastern region is higher than that of the central and western region. These results remain consistent after we experiment several robustness checks.

Findings in this paper carry broad implications. From the perspective of academic research, investigating the direct and moderating effects of environmental regulation intensity on enterprise technological innovation simultaneously and incorporating government financial support into the model as an important factor provide new insights into develop theories of environmental policy and management. For example, in the establishment and implement of China’s the environmental protection policy, which is more important: environmental regulation or government financial support? Whether there is interaction effects between environmental regulations and government financial support policies？How do government departments formulate environmental regulations and financial support policies to improve enterprise innovation? This study provides empirical evidence for better understand – and predict – the relationship of environmental regulation intensity, government financial support, and regional enterprise technological innovation to help guide the research in this filed.

From the perspective of the public policy-making, the research results of this paper show that there exists a U-shaped relation between environmental regulation intensity and technological innovation of enterprises which declines first and then climbs up. But government financial support does not significantly work on technological innovation directly, while environmental regulation drives this effect to be achieved, which highlights the complementarity between environmental regulation and government financial support. It suggests that enacting environmental regulation is the first step, but whether Chinese government can provide an effective incentive mechanism to firms to stimulate their technological innovation practices is another matter (Zhu et al., 2012, Guo, Qu, & Tseng, 2017). Hence, in order to change this relationship, it is necessary for Chinese government to adopt a hybrid approach combining government financial support and environmental regulation to motivate regional and enterprises’ technological innovation. Moreover, the government should further enhance environmental regulation intensity. On the one hand, it could force enterprises to apply technological innovation in pollution control and achieve pollution reduction and control in a high level. On the other hand, it could incentive enterprises to beef up innovation in production techniques and management methods (such as green human resource management), which is benefit for improving their productivity and international competitiveness (Yong, Yusliza, Ramayah, & Fawehinmi, 2019; Yong, Yusliza, Ramayah, & et al., 2019). But the government should also be alarmed that intensive environmental regulation may not be suitable to all region. Instead, the government ought to, based on the actual condition of specific region and sector, implement targeted and differentiated environmental regulation and make timely adjustments, rather than insist on a fixed standard, toward a proper range so as to keep the enabling effects of environmental regulation working (Porter, 1995).

From the perspective of the policy management and operational level, the government should identify the appropriate range for subsidy, but it should be considered synthetically from the whole angle, and adjusted dynamically at any time. Due to the positive externality of innovation and the crowding-out of R&D expense by environmental regulation, lack of funding in technological innovation is natural. In order to promote the interaction effects of environmental regulation and government financial support on enterprise technological innovation, the government should complement environmental regulation through reasonable funding and policy support. But this does not mean that the government should provide as much subsidy as possible. There exists a threshold value and proper range for environmental regulation to work on technological innovation effectively. When environmental regulation passes the inflection point and begins to enable technological innovation, government subsidy focusing on technological innovation can be cut down or even lifted.

Moreover, following your valuable suggestions, we have added some limitations and suggestions for future research. (1) Limitations in the measurement of variables: Existing studies on the relationship between environmental regulation and enterprise technological innovation in China have failed to effectively control for the impact of government financial support because of data limitations. For this research, a government financial support variable, based on the government-financed fund in R&D expense data, is developed and incorporated into the model, and while this variable is relatively consistent with the overall government financial support, there are still cannot distinguish whether it is for environmental-friendly technologies or not. In follow-up studies, additional data on government funding for green technologies need to be compiled, and combined with green innovation for further analysis. (2) Environmental regulations can be further subdivided: For example, it is possible to decompose environmental regulations into different sub-components based on type of regulation, such as the formal and informal environmental regulation. Further, it is also possible that testing the effect of environmental regulations against a wide range of different measures of technological innovation. However, due to manufacturing industry and regional panel data limitations, we are not yet able to examine this issue more extensively. The resulting problems need to be explored and new solutions developed in follow-up research. Therefore, in the latter study, we will try to use micro-enterprise level data to study the interaction effects of environmental regulation and government funding for green technologies on green innovation behavior of enterprises, and consider that in the different types of environmental regulation.

We greatly appreciate your comments and suggestions that are all valuable and very helpful for revising and improving the quality of our paper.

Finally, we tried our best to improve the manuscript and made some changes to the manuscript. These changes do not influence the context or framework of the paper. Although we did not list these changes here, they are shown in red in the revised manuscript.

We earnestly appreciate the editor’s and reviewers’ conscientious work and hope that the corrections will be met with approval.

Once again, thank you very much for your comments and suggestions.

References:

Acemoglu, D., Aghion, P., Bursztyn, L., & Hemous, D. (2012). The environment and directed technical change. American Economic Review, 102(1), 131-66.

Aghion, P., Dechezleprêtre, A., Hemous, D., Martin, R., & Van Reenen, J. (2016). Carbon taxes, path dependency, and directed technical change: Evidence from the auto industry. Journal of Political Economy, 124(1), 1-51.

Barbera, A. J., & McConnell, V. D. 1990. The impact of environmental regulations on industry productivity: direct and indirect effects. Journal of Environmental Economics and Management, 18(1), 50-65.

Berman, E., & Bui, L. T. (2001). Environmental regulation and productivity: evidence from oil refineries. Review of Economics and Statistics, 83(3), 498-510.

Brännlund, R., Färe, R., & Grosskopf, S. 1995. Environmental regulation and profitability: an application to Swedish pulp and paper mills. Environmental and Resource Economics, 6(1), 23-36.

Broekel, T., Brachert, M., Duschl, M., & Brenner, T. (2017). Joint R&D subsidies, related variety, and regional innovation. International Regional Science Review, 40(3), 297-326.

Bronzini, R., & Piselli, P. (2016). The impact of R&D subsidies on firm innovation. Research Policy, 45(2), 442-457.

Chen, B., & Feng, Y. (2000). Determinants of economic growth in China: Private enterprise, education, and openness. China Economic Review, 11(1), 1-15.

Clausen, T. H. (2009). Do subsidies have positive impacts on R&D and innovation activities at the firm level?. Structural change and economic dynamics, 20(4), 239-253.

Czarnitzki, D., & Lopes-Bento, C. (2014). Innovation subsidies: Does the funding source matter for innovation intensity and performance? Empirical evidence from Germany. Industry and Innovation, 21(5), 380-409.

Dinda, S. (2004). Environmental Kuznets curve hypothesis: a survey. Ecological economics, 49(4), 431-455.

Doloreux, D. (2002). What we should know about regional systems of innovation. Technology in society, 24(3), 243-263.

Levinson, A. (1996). Environmental regulations and manufacturers' location choices: Evidence from the Census of Manufactures. Journal of public Economics, 62(1-2), 5-29.

Li, G. S., & Shen, K. R. (2011). Study on Technological Introduction, Independent R&D and Innovation Performance in China. Journal of Finance and Economics, 11, 39-49.

Féres, J., & Reynaud, A. (2012). Assessing the impact of formal and informal regulations on environmental and economic performance of Brazilian manufacturing firms. Environmental and Resource Economics, 52(1), 65-85.

Geels, F. W. (2002). Technological transitions as evolutionary reconfiguration processes: a multi-level perspective and a case-study. Research policy, 31(8-9), 1257-1274.

Gemünden, H. G., Heydebreck, P., & Herden, R. (1992). Technological interweavement: a means of achieving innovation success. R&D Management, 22(4), 359-376.

Gollop, F. M., & Roberts, M. J. (1983). Environmental regulations and productivity growth: The case of fossil-fueled electric power generation. Journal of Political Economy, 91(4), 654-674.

Görg, H., & Strobl, E. (2007). The effect of R&D subsidies on private R&D. Economica, 74(294), 215-234.

Guellec, D., & Van Pottelsberghe de la Potterie, B. (1997). Does government support stimulate private R&D?. OECD economic studies, 95-122.

Guo, D., Guo, Y., & Jiang, K. (2016). Government-subsidized R&D and firm innovation: Evidence from China. Research policy, 45(6), 1129-1144.

Guo, L. L., Qu, Y., & Tseng, M. L. (2017). The interaction effects of environmental regulation and technological innovation on regional green growth performance. Journal of cleaner production, 162, 894-902.

Guo, J. (2019). The Effects of Environmental Regulation on Green Technology Innovation —— Evidence of the Porter Effect in China. Finance & Trade Economics, (3), 147-160. (In Chinese)

Gray, W. B. 1987. The cost of regulation: OSHA, EPA and the productivity slowdown. The American Economic Review, 77(5), 998-1006.

Greve, H. R. (2003). A behavioral theory of R&D expenditures and innovations: Evidence from shipbuilding. Academy of management journal, 46(6), 685-702.

Hamamoto, M. (2006). Environmental regulation and the productivity of Japanese manufacturing industries. Resource and Energy Economics, 28(4), 299-312.

Jaffe, A. B., Peterson, S. R., Portney, P. R., & Stavins, R. N. (1995). Environmental regulation and the competitiveness of US manufacturing: what does the evidence tell us?. Journal of Economic literature, 33(1), 132-163.

Jaffe, A. B., & Palmer, K. (1997). Environmental regulation and innovation: a panel data study. Review of Economics and Statistics, 79(4), 610-619.

Jia, P., Li, K., & Shao, S. (2018). Choice of technological change for China's low-carbon development: Evidence from three urban agglomerations. Journal of Environmental Management, 206, 1308-1319.

Jones, C. I. (2003). Human capital, ideas and economic growth. In Finance, Research, Education and Growth (pp. 51-74). Palgrave Macmillan, London.

Jorgenson, D. W., & Wilcoxen, P. J. (1990). Environmental regulation and US economic growth. The Rand Journal of Economics, 314-340.

Li, W., Gu, Y., Liu, F., & Li, C. (2018). The effect of command-and-control regulation on environmental technological innovation in china: a spatial econometric approach. Environmental Science and Pollution Research, (5), 1-12.

Magat, W. A. 1978. Pollution control and technological advance: A dynamic model of the firm. Journal of Environmental Economics and Management, 5(1), 1-25.

Malesky, E. J. (2008). Straight ahead on red: how foreign direct investment empowers subnational leaders. The Journal of Politics, 70(1), 97-119.

Palmer, K., Oates, W. E., & Portney, P. R. 1995. Tightening environmental standards: the benefit-cost or the no-cost paradigm?. Journal of Economic Perspectives, 9(4), 119-132.

Peng, B., Tu, Y., Elahi, E., & Wei, G. (2018). Extended Producer Responsibility and corporate performance: Effects of environmental regulation and environmental strategy. Journal of environmental management, 218, 181-189.

Porter, M. E. (1991). America’s green strategy, Scientific American, April. p. 96.

Porter, M. E., & Van der Linde, C. (1995). Toward a new conception of the environment-competitiveness relationship. Journal of economic perspectives, 9(4), 97-118.

Tang, J., Zhong, S., & Xiang, G. (2019). Environmental Regulation, Directed Technical Change, and Economic Growth: Theoretic Model and Evidence from China. International Regional Science Review, 0160017619835901.

Triebswetter, U., & Hitchens, D. (2005). The impact of environmental regulation on competitiveness in the German manufacturing industry—a comparison with other countries of the European Union. Journal of Cleaner Production, 13(7), 733-745.

Tsai, W. H., Lin, S. J., Liu, J. Y., Lin, W. R., & Lee, K. C. (2011). Incorporating life cycle assessments into building project decision-making: an energy consumption and co emission perspective. Energy, 36(5), 3022-3029.

Tsai, W. H., Lee, K. C., & Liu, J. Y., et al. (2012). A mixed activity-based costing decision model for green airline fleet planning under the constraints of the european union emissions trading scheme. Energy, 39(1), 218-226.

Van Rooij, B., Fryxell, G. E., Lo, C. W. H., & Wang, W. (2013). From support to pressure: The dynamics of social and governmental influences on environmental law enforcement in Guangzhou City, C hina. Regulation & Governance, 7(3), 321-347.

Wallsten, S. J. (2000). The effects of government-industry R&D programs on private R&D: the case of the Small Business Innovation Research program. The RAND Journal of Economics, 82-100.

Wang, H. (2002). Pollution regulation and abatement efforts: evidence from China. Ecological Economics, 41(1), 85-94.

Yang L. G., Gong S. H., Wang B., Chao Z. S. 2018. Human Capital, Technology Progress and Manufacturing Upgrading. China Soft Science, 1, 138-148

Yin, J., Zheng, M., & Chen, J. (2015). The effects of environmental regulation and technical progress on CO2 Kuznets curve: An evidence from China. Energy Policy, 77, 97-108.

Yong, J. Y., Yusliza, M-Y., Ramayah, T., & Fawehinmi, O. (2019). Nexus between green intellectual capital and green human resource management, Journal of Cleaner Production, 215, 364-374.

Yong, J. Y., Yusliza, M-Y., Ramayah, T., Jabbour, C. J. C., Sehnem, S. & Venkatesh M. (2019). Pathways towards Sustainability in Manufacturing Organizations: Empirical Evidence on the Role of Green Human Resource Management. Business Strategy and The Environment. Forthcoming.

Yu, F., Guo, Y., Le-Nguyen, K., Barnes, S. J., & Zhang, W. (2016). The impact of government subsidies and enterprises’ R&D investment: A panel data study from renewable energy in China. Energy Policy, 89, 106-113.

Zhang, J., Geng, H., Xu, G., et al. Research on the influence of environmental regulation on green technology innovation. China population, resources and environment, 2019, 29(1), 168-176. (In Chinese)

Zhang, Y., Wang, J., Xue, Y., & Yang, J. (2018). Impact of environmental regulations on green technological innovative behavior: An empirical study in China. Journal of Cleaner Production, 188, 763-773.

Zhao, X. , & Sun, B. . (2015). The influence of chinese environmental regulation on corporation innovation and competitiveness. Journal of Cleaner Production, 112(4), 1528-1536.

Zhou, Y., Zhu, S., & He, C. (2017). How do environmental regulations affect industrial dynamics? Evidence from China’s pollution-intensive industries. Habitat International, 60, 10-18.
